# Garden classification of femoral neck fracture using deep-learning algorithm

**DOI:** 10.1038/s41598-025-27766-4

**Published:** 2025-12-16

**Authors:** Jaebeom Yang, Jinyong Park, Keunwoo Park, Eic Ju Lim, Ji Wan Kim, Jihoon Kweon, Chul-Ho Kim

**Affiliations:** 1 Department of Biomedical Engineering, Asan Medical Center, University of Ulsan College of Medicine, 88 Olympic-ro 43-gil, Songpa-gu, Seoul, Republic of Korea; 2 Department of Orthopedic Surgery, Asan Medical Center, University of Ulsan College of Medicine, 88 Olympic-ro 43-gil, Songpa-gu, Seoul, Republic of Korea; 3 Department of Orthopedic Surgery, Chungbuk National University Hospital, Chungbuk National University College of Medicine, Cheongju, Republic of Korea

**Keywords:** Deep learning, Femoral neck fracture, Garden classification, X-ray, Musculoskeletal system, Engineering

## Abstract

The Garden classification, based on X-ray interpretation and established over 50 years ago, remains the standard clinical classification system for femoral neck fractures (FNFs). Yet, this classification has a high interobserver variability of 70%. We sought to develop a deep-learning algorithm capable of accurately predicting FNF types, using only X-ray images, with performance comparable to that of computed tomography (CT). We retrospectively collected data from 1,588 patients who underwent X-ray and 3D-CT scans and were diagnosed with femoral neck fractures at Asan Medical Center. The input X-ray dataset consisted of paired X-ray images of the hip, with anteroposterior (AP) and lateral views. Using 3D-CT as the reference standard, patients were labeled as Garden types I (*n* = 378, 23.8%), II (*n* = 68, 4.3%), III (*n* = 477, 30.0%), and IV (*n* = 665, 41.9%). Our algorithm consisted of hip-joint detection followed by Garden classification, for which 12 different deep-learning architectures were evaluated. Algorithm performance was externally validated in 100 patients. Our algorithms showed a 90.6% overall accuracy and 88.6% Dice similarity coefficient, indicating excellent FNF type discernment. Our algorithms could serve as a valuable tool for diagnosing FNF based on X-ray data only, with accuracy comparable to that of CT.

**Supplementary Information:**

The online version contains supplementary material available at 10.1038/s41598-025-27766-4.

## Introduction

Femoral neck fractures (FNFs) represent a significant orthopedic emergency, particularly among older individuals, and their accurate and timely diagnosis is critical for optimal patient outcomes. Typically, in cases of recent hip fracture, surgery is the consensus treatment^1,2^. Two mainstream treatment options are: osteosynthesis and arthroplasty, and these are further divided based on the type of fracture.

Usually in an emergency setting, clinicians face the challenge of promptly identifying and classifying hip fractures in a demanding and high-stress environment. A review study from the USA reported that misdiagnosis rates for hip fractures range from 2% to 10%^3^, emphasizing the need for reliable screening tools.

Despite being in use for over 60 years, the Garden classification system remains the most widely utilized method globally for classifying FNFs in a clinical setting. The Garden classification system is advantageous due to its simplicity and intuitive nature. However, it has clear limitations, primarily because it defines fracture types solely on anteroposterior (AP) radiographs. However, most FNFs require evaluation using radiographs in multiple planes or even with advanced imaging. Advances in computed tomography (CT) have shown that many FNFs that are classified as type I on plain radiographs may potentially be Garden types III or IV displaced fractures^4^. Moreover, the Garden classification based on AP radiographs alone has been reported to have an interobserver kappa value in the range of only 0.03–0.56, indicating low reliability^4,5^. Intraobserver reliability has also been reported to be relatively low, with a kappa value of approximately 0.759^4,6^. Nevertheless, advanced imaging tools, such as CT, which may provide more reliable categorization, are unfortunately not available in all clinical settings.

The potential of artificial intelligence (AI) as a transformative tool in medical diagnosis and fracture classification has garnered attention. By automating the classification process, AI has the capacity to reduce the time and effort required by medical staff in various tasks, and can demonstrate accuracy beyond human capabilities, even with limited information^7^. Moreover, AI-driven diagnostics offer the promise of improving diagnostic accuracy and consistency, thereby enhancing patient outcomes, and reducing the risk of misdiagnosis.

We sought to facilitate screening of fracture types without incurring the medical costs and radiation harm associated with CT scans. To this end, we developed an AI-based screening algorithm for FNF classification that can accurately predict CT-based diagnoses using only X-ray images. We also proposed a method to identify patient groups with high predictive accuracy using confidence, defined as the prediction probabilities of deep learning models.

## Methods

This study was approved by the Ethics Committee of Institutional Review Board of Asan Medical Center, Seoul, Republic of Korea (IRB No. 2023 − 0517), which waived the need to obtain informed consent due to the retrospective nature of the study and the use of anonymous clinical data in the analysis. Data collection was performed in accordance with the relevant guidelines and regulations of the committee.

### Research participants and data collection

We retrospectively reviewed the consecutive clinical data of patients who were diagnosed with FNF at the Asan Medical Center between January 2003 to April 2023. We included the data of the patients who had hip X-rays of both the AP and lateral (LA) views, and who underwent preoperative 3D-CT scans. All X-rays and CT scans used in the study were taken on the same day or within one day of the patient’s hospital admission for FNF. We excluded pediatric cases where the growth plates had not closed, and cases where the quality of the X-rays was inadequate or where the CT scans were taken at a lower resolution at an outside hospital. We did not exclude patients who had vascular calcification or a Foley catheter on X-ray, and we also did not consider the position of the hip joint on the X-ray as an exclusion criterion affecting the quality assessment of the hip simple radiograph, as these were regarded as normal variations in real-world data. After applying these inclusion and exclusion criteria, the data of 1,588 patients were finally included in the internal dataset. The demographics of internal dataset including patient age, sex, fracture type are shown in Table 1.

**Table 1:** Comparison of demographics between internal and external Datasets.

	Internal dataset (*n* = 1,588)	External dataset (*n* = 100)	*P*-value
Age, yrs	72.8 ± 12.8 (range, 19–102)	75.1 ± 13.8 (range, 28–99)	0.107
Female, n	1,111 (70.0%)	70 (70%)	1.000
Garden type, n			0.032
I	378 (23.8%)	24 (24%)	
II	68 (4.3%)	10 (10%)	
III	477 (30.0%)	22 (22%)	
IV	665 (41.9%)	44 (44%)	

### Data labeling: X-ray and 3D-CT imaging

**Fig. 1:**
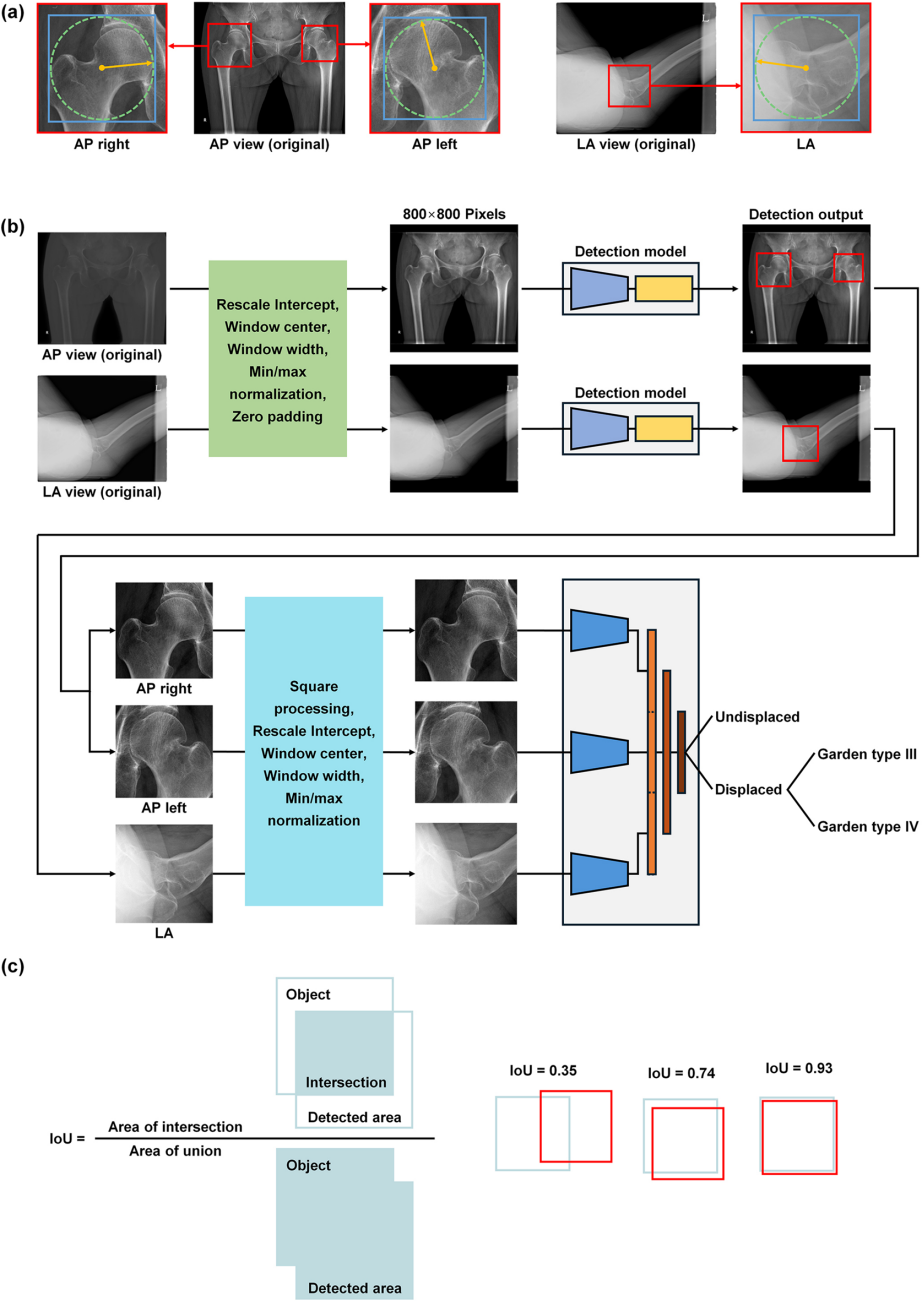
(a) Bounding box labeling for hip-joint detection. (b) Schematic diagram of deep-learning networks for hip-joint detection and femoral neck fracture classification. (c) Definition of evaluation metrics for hip-joint detection and examples. AP, anteroposterior; LA, lateral.

The deep-learning approaches in this study consisted of two stages: detection of the femoral neck in X-ray images and classification of the Garden type using the detected areas (Fig. 1a). For the first stage of detection, the hip joint was labeled using a bounding box (Fig. 1b). A circle was created with its center at the middle of the femoral neck and its radius extending to the farthest point of the femoral head, with a margin of 50 pixels. A square that encompasses this circle was set as the ground truth. This method was applied consistently to both the AP and LA views, resulting in three labels for each patient: two for the AP view (one each for the fracture site and the unaffected side) and one for the LA view.

**Fig. 2:**
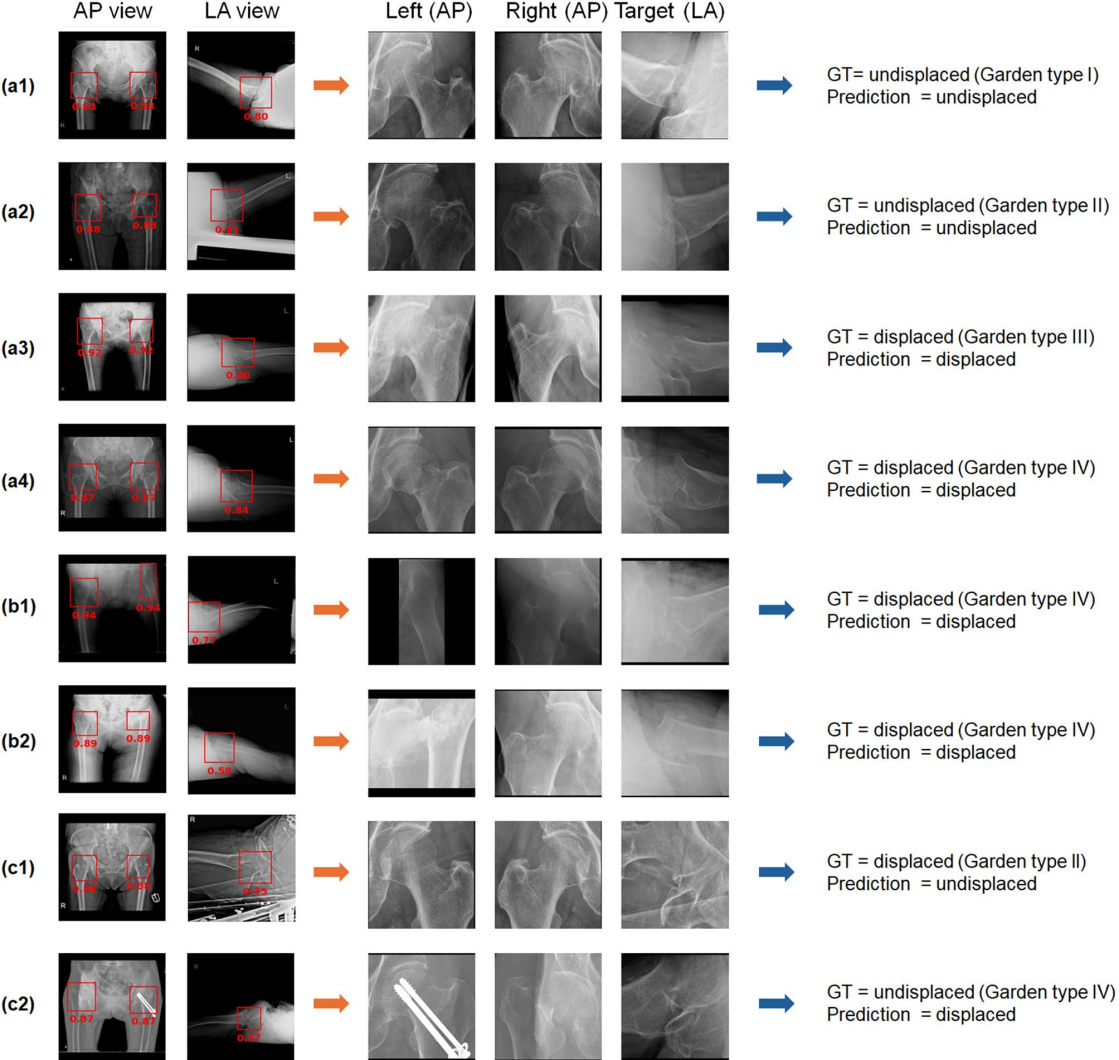
Representative examples of detection and classification models for femoral neck fracture: (a1–a4) Correct detections and classifications; (b1, b2) Cases with left hip-joint undetected in the anteroposterior (AP) view; (c1, c2) cases with hip-joint undetected in the lateral (LA) view. Detected bounding boxes and corresponding intersection over union (IoU) values are indicated in red. GT, ground truth.

For the second stage of the Garden classification, 3D-CT was used to determine the ground truth, with a single label assigned per patient referring to the more severely affected hip joints. For example, when Garden type II was observed on the left side and Garden type III on the right side, Garden type III was assigned as the final label. Two experienced orthopedists independently classified each case on 3D-CT and when labels were discrepant, they determined the final label by discussion to agreement. Accordingly, internal dataset was composed of Garden types I (*n* = 378, 23.8%), II (*n* = 68, 4.3%), III (*n* = 477, 30.0%), and IV (*n* = 665, 41.9%).

### Deep-Learning networks

For the first stage of deep-learning detection, a Faster R-convoluted neural network (CNN) was used to identify the hip joint in X-ray images (Fig. 1b)^8^. Original X-ray images varied in format (1070–3520 × 1430–4280 pixels) depending on the patient and imaging device. These images were resized with the longer dimension fixed at 800 pixels, followed by min/max normalization. The images were then zero-padded to create 800 × 800-pixel dimensions to be used as network inputs (Fig. 1b). Separate networks were configured for detection in the AP and LA views, and three areas were ultimately output in the detection stage.

In the second stage of deep-learning classification, the images corresponding to the three areas acquired in the detection stage were used as input. If the image was not square, it was expanded in the short-axis direction by using information from the original X-ray image. Input images, resized as 256 × 256, were normalized by using min/max normalization. Images were used to train a separate network and were combined in the final three fully convolutional layers to determine the final output. The initial weight was adopted from ImageNet for transfer learning. Weighted binary cross-entropy loss was applied as the loss function, defined as:

1 $${\text{Loss}} = - \sum {\left[ {w \cdot y \cdot \log (p) + (1 - y) \cdot \log (1 - p)} \right]}$$

where w is the weight for the positive class, y is the ground truth label, and p is the predicted probability. The weight w was determined based on the inverse frequency of each class to compensate for class imbalance.

### Training setup

The deep-learning models for detection and classification were trained for a maximum of 100 and 200 epochs, respectively, with an initial learning rate of 10^− 3^. The AdamW optimizer was applied with β1 = 0.9 and β2 = 0.999. The mini-batch size was adjusted to produce better outcomes (8 for detection and 32 for classification). For classification, only horizontal flip was applied in the data augmentation process. Further details of the training setup can be found in Supplementary Table 1. The deep-learning networks were implemented using PyTorch in Python and were trained on a workstation with an Intel Core i9-10920X processor and four NVIDIA GeForce RTX 2080 Ti graphics processing units.

To compare the performance of deep-learning models, five-fold cross-validation was applied, ensuring equal distribution of images from each Garden type across all folds. In the cross-validation, the fold-proportion of training, validation, and test sets was 3:1:1, and the fold-composition was changed in sequence under cyclic permutation.

### Performance evaluation

Detection performance was evaluated using precision, recall, intersection over union (IoU), and mean average precision (mAP). Precision was defined as $\:Pr\:=\:TP/\left(TP+FP\right)$ and recall as $\:Re\:=\:TP/\left(TP+FN\right)$, where TP is true-positive, FP is false-positive, and FN is false-negative. When IoU was greater than a threshold of 0.5 in this study (Fig. 1c), the prediction was considered TP. mAP was calculated by averaging the area under the precision-recall curve (AUC) across all object classes.

For classification, 12 networks were evaluated. For further improvement of the classification performance, ensemble learning with hard voting was performed using the three best-performing models based on the Dice similarity coefficient (DSC). DSC, also known as the F1-score in machine learning, was defined as:

2 $$\:DSC\:=\:\frac{2\times\:TP}{2\times\:TP+FN+FP}\:=\:\frac{2\times\:Pr\times\:Re}{Pr+Re}$$

To address the data imbalance issue, we first classified the fractures as undisplaced (Garden types I and II) versus displaced (Garden types III and IV) fractures, according to the simplified Garden classification^9,10^, and then performed additional classification into Garden types III and IV for cases predicted as displaced (Fig. 1b). The flow from ground truth to prediction outcomes was visualized with Sankey diagrams.

To evaluate whether the information from the LA view could improve classification performance, we compared the prediction outcomes to those using information of only the AP view. Finally, we proposed a method for identifying patient groups with high predictive accuracy using confidence (prediction probabilities). The evaluation metrics for classification were accuracy, precision, recall, and DSC.

### External validation

For external validation, anonymized data from 100 patients with FNFs, treated between January 2019 and April 2022, were obtained from Chungbuk National University Hospital. Data labeling was initially performed by an orthopedic professor with expertise in FNFs at Chungbuk National University Hospital (E. J. Lim) and was validated by an orthopedist at Asan Medical Center (C. H. Kim). The external dataset was evaluated using five algorithm sets, each trained on different fold-combinations from the internal dataset during cross-validation, as described earlier, and the average performance was reported. Otherwise, the external dataset was evaluated in the same manner as was the internal dataset. No additional training was performed using the external data. Using 3D-CT as the reference standard, patients were labeled as Garden types I (*n* = 24, 24%), II (*n* = 10, 10%), III (*n* = 22, 22%), and IV (*n* = 44, 44%) in external dataset.

### Code Availability

All custom code used in this study is openly available under Apache License 2.0 at https://github.com/AMC-CBN/FNF_OPEN_REPO. The repository provides environment files with pinned dependencies and step-by-step instructions in the README to reproduce data preprocessing, model training, and inference. Complete training and inference scripts, model architectures, and hyperparameters are included to enable faithful re-training once appropriate data access is obtained through the controlled process described above. Trained model weights derived from protected clinical data are not publicly distributed due to privacy and institutional restrictions.

## Results

### Cross-Validation in the internal dataset

#### Detection of the hip joint

The hip joint was accurately detected by the algorithms, with precision and recall ranging from 98.5% to 100% (Table 2; Fig. 2a1–2a4). In the AP view, only two images had the left hip joint categorized as undetected (Fig. 2b1, b2). Even in these cases, the FNF was still included in the predicted area. The average IoUs for the left and right hip joints were 86.1%. In the LA view, low contrast around the femoral neck led to 24 of 1,588 images being categorized as undetected, of which Garden type I accounted for 4 cases, type III for 8 cases, and type IV for 12 cases (Fig. 2c1, c2). However, of these, fracture site capture failed in only seven images (0.4%).

**Table 2:** Detection performance of deep-learning models for the hip joint in terms of precision, recall, intersection over union (IoU), and mean average precision (mAP).

Target	Precision (%)	Recall (%)	IoU (%)	mAP (%)
Internal dataset (*N* = 1,588)				
AP view—Left hip joint	99.9	99.9	86.1	99.9
AP view—Right hip joint	100	100	86.1	100
LA view	98.5	98.5	79.4	98.3
External dataset (*N* = 100)				
AP view—Left hip joint	99.4	99.2	77.2	99.2
AP view—Right hip joint	99.8	99.6	82.2	99.6
LA view	84.0	84.0	67.4	79.5

N, number of patients; AP, anteroposterior; LA, lateral.

#### Classification of undisplaced (Garden type I & II) versus displaced (Garden type III & IV) fractures

**Fig. 3:**
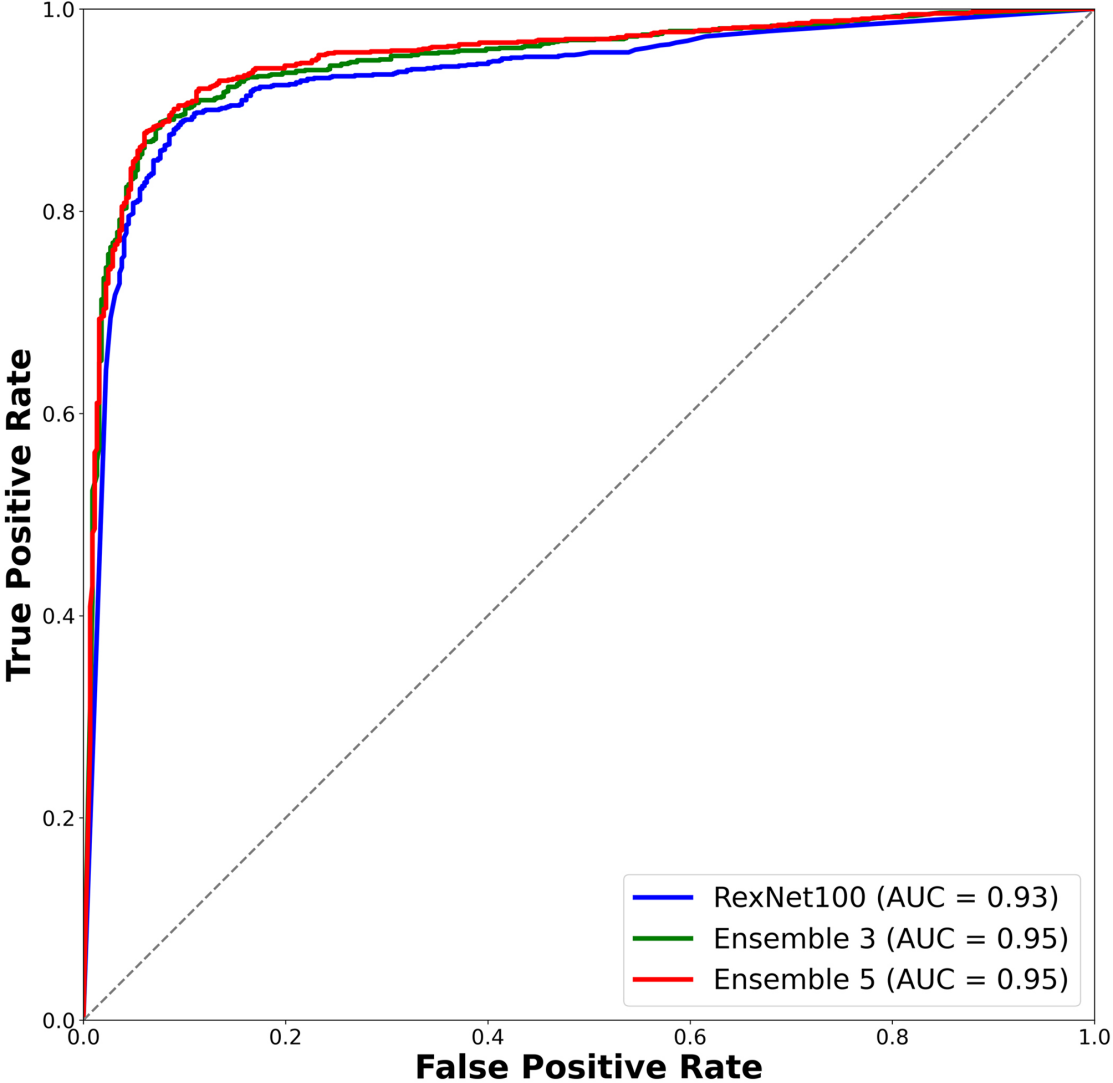
Receiver operating characteristic curves for femoral neck fracture classification using deep-learning models: undisplaced (Garden types I and II) versus displaced (Garden types III and IV). The ensemble result was obtained by combining outputs from MobileNetV3, EfficientNetB4, and ResNet18. DSC, Dice Similarity Coefficient; AUC, area under curve.

When using both the AP and LA views for prediction, the classification performance achievable with a single deep-learning network ranged from 87.2% to 89.6% for accuracy and from 84.1% to 87.3% for DSC (Table 3). The performance was relatively better for the displaced (Garden type III and IV) fractures, which included more cases, with maximum precision and recall values of 94.8% and 91.9%, respectively. Based on the DSC, MobileNetV3 showed the best performance (AUC = 0.93), followed by EfficientNetB4, and ResNet18. When the ensemble approach was applied, the DSC reached 88.6% (AUC = 0.95), showing improvement of 1.3% over the best-performing single network (Fig. 3). For the 24 cases classified as undetected in the LA view, the four models (MobileNet, ResNeXt50, RexNet100, and RexNet130) correctly predicted 22 cases, and in the remaining cases, they achieved correct predictions in 23 cases. The accuracy, AUC, and DSC for each fold are summarized in Supplementary Table 2.

**Table 3:** Classification performance of deep-learning models for femoral neck fracture: undisplaced (Garden types I and II) versus displaced (Garden types III and IV) in cross-validation using the internal dataset (*N* = 1,588). For individual networks, the best performance value for each metric is highlighted in bold. An ensemble approach was applied in order of ranking based on DSC. For the model using both anteroposterior (AP) and lateral (LA) views, MobileNetV3, EfficientNetB4, and ResNet18 were the top 3 algorithms.

Name	Overall						Undisplaced (Garden type I and II)				Displaced (Garden type III and IV)			
	ACC (%)		AUC (%)		DSC (%)		Pr (%)		Re (%)		Pr (%)		Re (%)	
	AP	AP + LA	AP	AP + LA	AP	AP + LA	AP	AP + LA	AP	AP + LA	AP	AP + LA	AP	AP + LA
EfficientNetB0	88.5	88.4	**93.5**	93.7	86.3	85.9	76.3	77.6	85.9	82.5	94.2	93.0	89.6	90.7
EfficientNetB2	87.8	88.4	92.5	93.0	85.1	85.8	77.2	78.5	80.5	80.9	92.3	92.5	90.7	91.3
EfficientNetB4	88.4	89.2	92.8	93.3	86.0	86.8	77.2	79.1	83.4	83.4	93.3	93.4	90.4	91.4
ResNet18	86.3	88.6	92.8	93.5	83.6	86.5	73.1	75.7	81.2	**87.4**	92.3	**94.8**	88.4	89.1
ResNet50	87.5	88.5	92.6	**94.2**	85.0	86.0	75.5	77.7	82.3	82.7	92.8	93.1	89.6	90.7
ResNet101	88.0	88.5	93.2	93.6	85.4	85.7	77.1	79.4	81.6	79.6	92.7	92.0	90.5	**91.9**
ResNext50	87.3	88.6	92.5	93.7	84.7	86.2	75.6	77.8	81.2	83.2	92.4	93.2	89.8	90.7
ReXNet100	88.6	88.6	93.0	93.7	86.3	86.5	77.2	75.8	84.3	87.2	93.6	94.7	90.3	89.1
ReXNet130	87.5	88.4	93.3	93.4	85.4	85.7	73.4	78.2	**87.2**	81.2	**94.6**	92.5	87.7	91.2
ReXNet150	**89.1**	88.4	93.2	94.0	**87.0**	85.7	**77.5**	78.3	86.3	80.9	94.4	92.5	90.2	91.2
DenseNet121	87.2	87.2	93.2	93.7	84.4	84.1	76.0	77.8	79.6	76.2	91.9	90.8	90.2	91.5
MobileNetV3	86.8	**89.6**	91.5	93.5	83.6	**87.3**	76.7	**80.1**	76.0	83.9	90.7	93.6	**91.0**	**91.9**
Ensemble of top 3	89.9	90.6	94.8	94.9	87.9	88.6	79.1	80.5	87.2	87.7	94.8	95.0	91.0	91.7

ACC, accuracy; AUC, area under the curve; DSC, Dice Similarity Coefficient; Pr, precision; Re, recall.

**Fig. 4:**
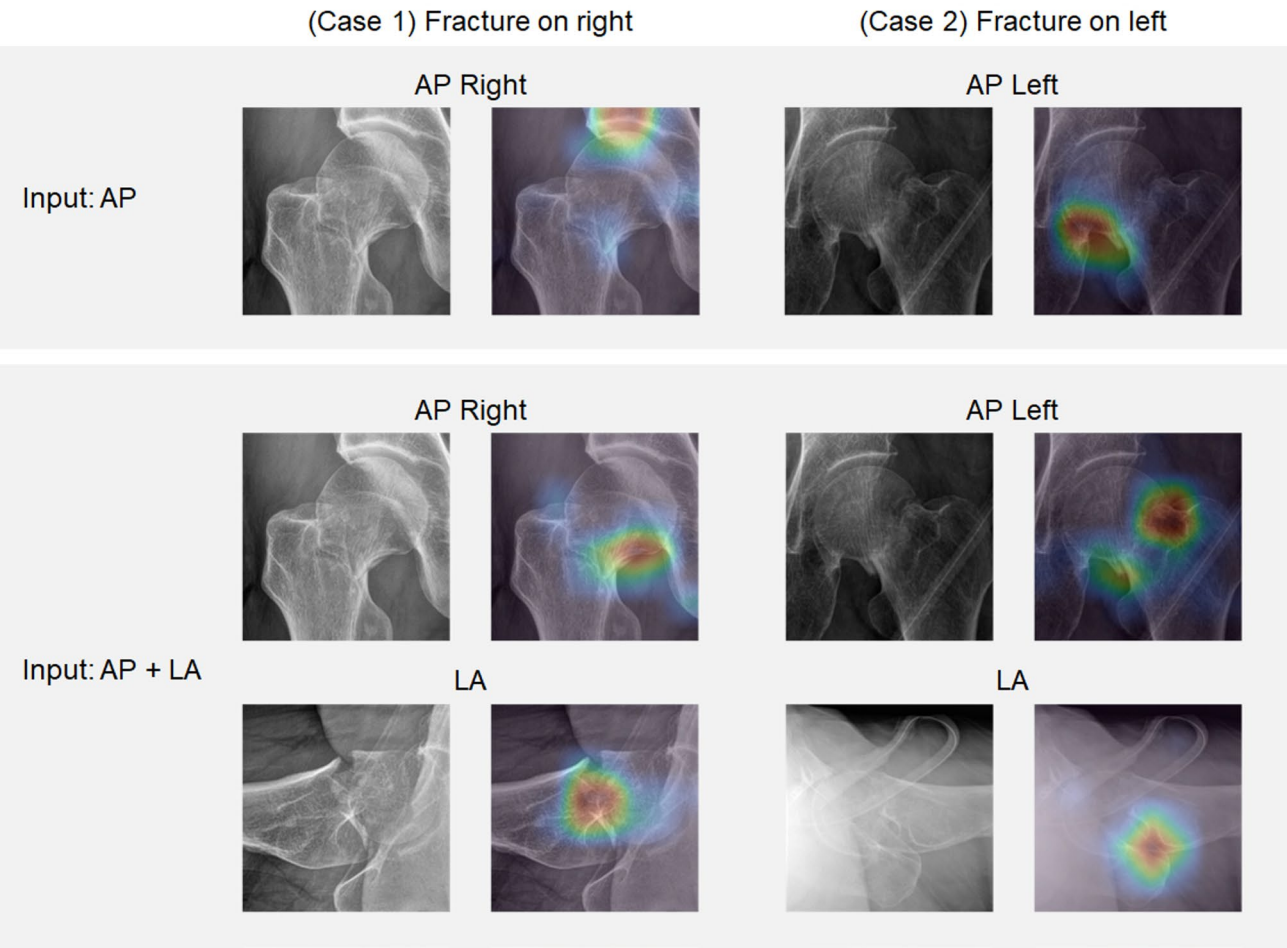
Case examples illustrating prediction differences between AP and AP + LA models visualized with LayerCAM (MobileNetV3). In these posterior tilt fracture cases, the AP model predicted the fractures as undisplaced, whereas the inclusion of lateral views as input enabled correct prediction as displaced fractures. AP, anteroposterior; LA, lateral.

Compared to using the AP view information alone, adding LA view information could increase the accuracy of deep-learning prediction by up to 2.8% and the DSC by up to 3.7%. As illustrated in the case examples (Fig. 4), models trained with AP views alone tended to misclassify posterior tilt fractures as undisplaced, whereas incorporating LA view information enabled correct prediction as displaced fractures. However, for EfficientNetB0 and ReXNet150 models, the accuracy was decreased slightly.

**Fig. 5:**
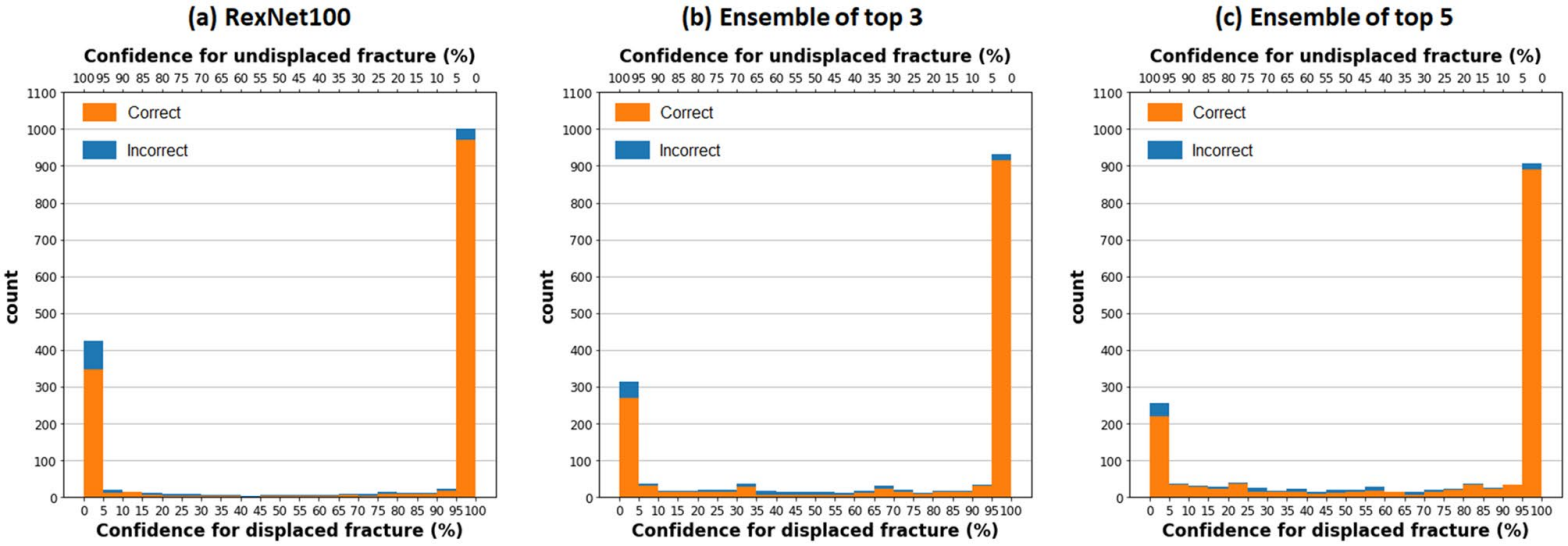
Distribution of patients according to confidence. Confidence indicates the predicted probability output by the deep-learning model. On the X-axis, confidence for displaced fractures (Garden types III and IV) increases to the right, while confidence for undisplaced fractures (Garden types I and II) increases to the left.

Figure 5 shows the distribution of prediction results according to confidence, defined as predicted probability. Most cases in this study were placed within the high confidence region ≥ 95%. The cases with high confidence (versus total patients) demonstrated improved accuracy of 3.2–5.4% and enhanced DSC of 3.3–5.9% (Table 4). MobileNetV3, the best-performing model in this study, achieved a diagnostic accuracy of 93.1% for 87.8% of all patients. Applying ensemble methods increased the diagnostic accuracy to over 95.4%, but 22.6% of patients fell into the gray zone, with confidence < 95%.

**Table 4:** Confidence-based identification of patient groups with high predictive performance for undisplaced (Garden types I and II) and displaced (Garden types III and IV) fractures in cross-validation using the internal dataset (*N* = 1,588). Confidence indicates the predicted probability output by deep-learning models. The Gray zone represents patient groups with confidence below 95% for both undisplaced and displaced fractures. For individual networks, the best performance value for each metric is highlighted in bold. The ensemble results were obtained by combining the outputs of MobileNetV3, EfficientNetB4, and ResNet18, which were the top 3 models ranked based on DSC in total.

Name	ACC (%)		AUC (%)		DSC (%)		Number of patients in gray zone
	Total	Confidence ≥ 95%	Total	Confidence ≥ 95%	Total	Confidence ≥ 95%	
EfficientNetB0	88.4	92.2	93.7	95.0	85.9	89.9	201 (12.7%)
EfficientNetB2	88.4	92.1	93.0	94.0	85.8	89.5	188 (11.8%)
EfficientNetB4	89.2	92.6	93.3	94.3	86.8	90.1	200 (12.6%)
ResNet18	88.6	91.8	93.5	94.5	86.5	89.9	173 (10.9%)
ResNet50	88.5	92.9	**94.2**	**95.6**	86.0	90.7	209 (13.2%)
ResNet101	88.5	92.4	93.6	94.9	85.7	89.8	206 (13.0%)
ResNext50	88.6	92.2	93.7	94.8	86.2	89.9	178 (11.2%)
ReXNet100	88.6	92.5	93.7	94.6	86.5	90.6	170 (10.7%)
ReXNet130	88.4	92.1	93.4	94.4	85.7	89.7	182 (11.5%)
ReXNet150	88.4	92.6	94.0	95.3	85.7	90.1	197 (12.4%)
DenseNet121	87.2	92.6	93.7	95.4	84.1	90.0	228 (14.4%)
MobileNetV3	**89.6**	**93.1**	93.5	94.4	**87.3**	**90.9**	194 (12.2%)
Ensemble of top 3	90.6	95.4	94.9	96.4	88.6	93.6	359 (22.6%)

ACC, accuracy; AUC, area under the curve; DSC, Dice similarity coefficient.

#### Classification of garden type III versus IV

The deep-learning models for classifying Garden type III and IV fractures showed a lower predictive capability than that of the previous stage of undisplaced versus displaced classification. Among single networks, EfficientNetB4 demonstrated the highest accuracy of 68.6%. The maximum DSC achievable using an ensemble of the top 3 models (ResNet101, EfficientNetB4, and ResNet50, in order of higher DSC) was 67.6% (Table 5). When limited to cases with confidence ≥ 95%, the DSC of individual networks could be improved to 70.6%, but this approach risked missing 37.7% of patients (Table 6). The outcome of the 5-fold cross-validation conducted exclusively on cases labeled as Garden type III and IV fractures is found in Supplementary Table 3.

**Table 5:** Classification performance of deep-learning models for femoral neck fracture: garden type III versus IV in cross-validation using the internal dataset (*N* = 1,588). These results include cases classified as displaced (Garden types III and IV) from the undisplaced (Garden types I and II) in the previous step. The cross-validation results of deep-learning models using only displaced (Garden types III and IV) cases can be found in supplementary table 2. For individual networks, the best performance value for each metric is highlighted in bold. The ensemble results were obtained by combining the outputs of ResNet101, EfficientNetB4, and ResNet50, which were the top 3 models ranked based on DSC.

Name	Overall			Garden type 3		Garden type 4	
	ACC (%)	AUC (%)	DSC (%)	Pr (%)	Re (%)	Pr (%)	Re (%)
EfficientNetB0	67.0	68.7	62.7	57.1	44.7	71.0	80.2
EfficientNetB2	67.2	71.0	63.6	56.9	48.1	71.9	78.5
EfficientNetB4	**68.6**	70.0	64.9	**59.5**	48.6	72.6	**80.5**
ResNet18	65.5	67.9	60.8	54.8	41.3	69.7	79.8
ResNet50	66.9	69.1	64.3	55.6	53.7	73.2	74.7
ResNet101	67.4	71.1	**65.5**	55.7	**59.4**	**75.0**	72.1
ResNext50	67.1	**71.2**	64.0	56.2	51.2	72.6	76.5
ReXNet100	63.4	65.1	60.0	50.9	45.7	69.7	73.9
ReXNet130	66.6	67.8	62.5	56.3	45.0	70.9	79.4
ReXNet150	67.9	70.4	63.9	58.4	46.8	71.9	80.3
DenseNet121	65.5	68.1	62.6	53.8	50.6	71.8	74.4
MobileNetV3	66.6	69.0	63.4	55.7	49.6	72.0	76.6
Ensemble of top 3	70.4	73.9	67.6	61.4	54.8	74.9	79.7

ACC, accuracy; AUC, area under the curve; DSC, Dice similarity coefficient; Pr, precision; Re, recall.

**Table 6:** Confidence-based identification of patient groups with high predictive performance for garden types III and IV in cross-validation using the internal dataset (*N* = 1,588). Confidence indicates the predicted probability output by deep-learning models. The Gray zone represents patient groups with confidence below 95% for both garden types III and IV. For individual networks, the best performance value for each metric is highlighted in bold. The ensemble results were obtained by combining the outputs of ResNet101, EfficientNetB4, and ResNet50, which were the top 3 models ranked based on DSC in total.

Name	ACC (%)		AUC (%)		DSC (%)		Number of patients in gray zone
	Total	Confidence ≥ 95%	Total	Confidence ≥ 95%	Total	Confidence ≥ 95%	
EfficientNetB0	67.0	70.7	68.7	69.0	62.7	63.2	305 (29.3%)
EfficientNetB2	67.2	71.7	71.0	**74.5**	63.6	66.4	279 (26.8%)
EfficientNetB4	**68.6**	74.1	70.0	70.0	64.9	66.7	431 (41.4%)
ResNet18	65.5	74.2	67.9	70.7	60.8	65.7	481 (46.2%)
ResNet50	66.9	73.1	69.1	72.2	64.3	69.0	395 (37.9%)
ResNet101	67.4	72.5	71.1	74.0	**65.5**	69.5	350 (33.6%)
ResNext50	67.1	**75.3**	**71.2**	74.2	64.0	**70.6**	393 (37.7%)
ReXNet100	63.4	69.0	65.1	66.4	60.0	63.1	336 (32.2%)
ReXNet130	66.6	72.2	67.8	68.8	62.5	65.0	373 (35.8%)
ReXNet150	67.9	75.0	70.4	71.8	63.9	68.9	366 (35.1%)
DenseNet121	65.5	72.8	68.1	70.5	62.6	67.3	427 (41.0%)
MobileNetV3	66.6	72.0	69.0	70.9	63.4	67.1	313 (30.0%)
Ensemble of top 3	70.4	81.5	73.9	74.2	67.6	73.3	691 (66.3%)

ACC, accuracy; AUC, area under the curve; DSC, Dice similarity coefficient.

#### Sankey diagram

**Fig. 6:**
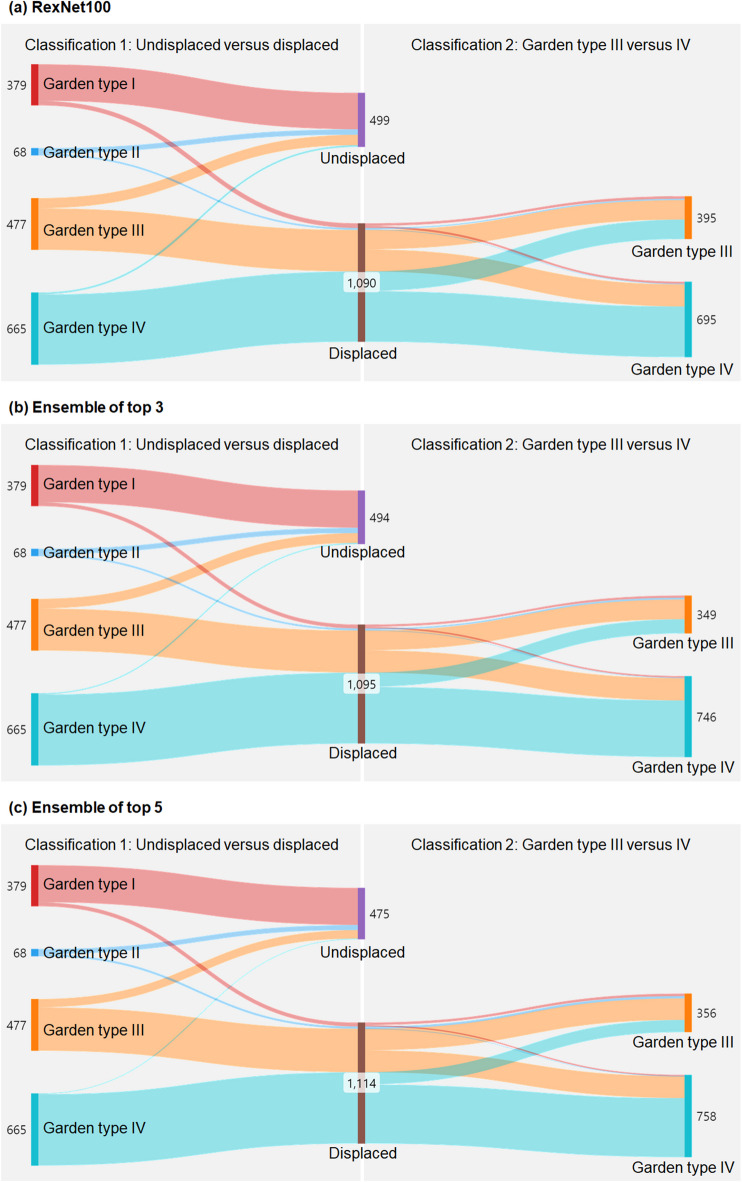
Sankey diagrams showing the flow from ground truth to prediction outcomes. The flows on the left side indicate deep-learning classification between undisplaced (Garden types I and II) and displaced (Garden types III and IV) fractures. The flows on the right side indicate the subsequent classification between Garden types III and IV fractures.

A Sankey diagram was used to visualize how each Garden type FNF was classified by the deep-learning models (Fig. 6). In the stage of distinguishing between undisplaced and displaced fractures, the risk was frequently underestimated by classifying displaced fractures as undisplaced fractures. Instances of misclassifying Garden type IV as undisplaced were exceedingly rare (Supplementary Fig. 1). In the differentiation between Garden types III and IV, a high proportion of incomplete displaced fractures (Garden type III) were erroneously identified as complete displaced fractures (Garden IV).

#### External validation

Precision and recall were greater than 99.2% for the left and right hip joints in the AP view, respectively, while decreasing by 14.5% in the LA view as compared to the internal cross-validation (Table 2). The LA view exhibited higher numbers of undetected images (1 vs. 16 on average, from cross-validation), with an average of 2.6 cases where the FNFs were outside the predicted bounding box.

The deep-learning networks achieved classification accuracy of 85.0–87.8% (Table 7). Based on the classification accuracy, EfficientNetB2 exhibited the least decrease (−0.6%) on external validation, while ReXNet130 showed the largest decrease (−3.4%). Similar to the internal validation results, displaced fractures (Garden type III and IV) were better classified than undisplaced fractures (Garden type I and II). Ensemble approaches improved the accuracy to 88.4%. The accuracy, AUC, and DSC of the five models are summarized in Supplementary Table 4.

**Table 7:** Classification performance of deep-learning models femoral neck fracture: undisplaced (Garden types I and II) versus displaced (Garden types III and IV) in external dataset (*N* = 100). Prediction models using both anteroposterior (AP) and lateral (LA) views were applied and the scores were averaged for five cross-validation models. For individual networks, the best performance value for each metric is highlighted in bold. The ensemble results were obtained by combining the outputs of MobileNetV3, EfficientNetB4, and ResNet18, which were the top 3 models ranked based on DSC in internal dataset.

Name	Overall			Undisplaced (Garden type I and II)		Displaced (Garden type III and IV)	
	ACC (%)	AUC (%)	DSC (%)	Pr (%)	Re (%)	Pr (%)	Re (%)
EfficientNetB0	86.8	92.6	85.8	76.0	89.4	94.0	85.5
EfficientNetB2	**87.8**	93.0	**87.0**	76.6	92.4	95.6	85.5
EfficientNetB4	87.6	92.6	86.8	76.0	92.9	95.9	84.8
ResNet18	85.4	91.1	84.5	73.0	90.6	94.5	82.7
ResNet50	87.4	92.4	86.6	75.4	**93.5**	**96.2**	84.2
ResNet101	86.8	93.1	85.8	76.3	88.8	93.7	85.8
ResNext50	87.0	**93.4**	86.0	76.4	89.4	94.0	85.8
ReXNet100	85.6	91.2	84.7	73.3	90.6	94.5	83.0
ReXNet130	85.0	90.8	84.0	73.2	88.2	93.2	83.3
ReXNet150	87.4	92.9	86.5	**76.9**	90.0	94.4	**86.1**
DenseNet121	85.4	92.2	84.2	75.4	84.7	91.6	85.8
MobileNetV3	86.4	91.8	85.4	75.2	89.4	94.0	84.8
Ensemble of top 3	88.4	93.0	87.7	76.9	94.1	96.6	85.5

ACC, accuracy; AUC, area under the curve; DSC, Dice similarity coefficient; Pr, precision; Re, recall.

## Discussion

In the current study, we developed an AI model capable of accurately detecting fracture sites from simple hip X-rays. We also developed a model that performs excellently in classifying FNFs into undisplaced and displaced types. Furthermore, within the displaced fracture type, we developed a model capable of screening for Garden types III or IV fractures, with acceptable performance. This suggests that, by utilizing deep-learning techniques, it is possible to achieve fracture classification accuracy comparable to that of CT scans, even in situations where obtaining appropriate data through CT imaging is not feasible.

Generally, surgical intervention is now established as the consensus primary approach for treating FNFs^11^. Surgical treatment can be categorized into two main options: osteosynthesis and arthroplasty. The femoral head receives its blood supply from three sets of vessels: the superior, inferior, and anterior retinacular arteries^12^. The extent of vascular damage is closely linked to the fracture displacement, which in turn affects the likelihood of femoral head osteonecrosis^13^. Therefore, the most important factor in choosing the appropriate surgical option between osteosynthesis and arthroplasty is the fracture pattern, specifically whether it is an undisplaced (Garden types I and II) or displaced type (Garden types III and IV). Undisplaced types are typically treated with osteosynthesis, while displaced types are treated with arthroplasty. Thus, fracture classification is crucial in determining the treatment direction. The screening tool developed in this study can significantly aid in determining the treatment direction, based on X-rays only.

The Garden classification has a long history of use and remains one of the most widely used classification systems for FNFs in clinical practice, due to its convenience. Introduced by British orthopedic surgeon Robert Symon Garden in 1961, this classification system divides FNFs into four groups based on fracture displacement, completeness, and the relationship of bony trabeculae in the femoral head and neck^4^. However, the very low interobserver reliability of the Garden classification is a significant drawback. The interobserver kappa value ranges between 0.03 and 0.56, indicating poor reliability^4^, particularly in terms of accurately reflecting posterior tilt. Therefore, caution must be exercised when planning osteosynthesis solely based on the appearance of nondisplaced fractures resembling Garden types I or II on simple hip AP X-ray images.

With the advancement of AI in medical imaging, several studies have applied AI techniques to perform Garden classification. Krogue et al.^14^ developed a deep learning model to classify hip fractures into six categories, including normal and previous arthroplasty, based on X-ray images from 1,118 patients. FNF types were categorized only as displaced or undisplaced, without individual Garden type classification. Undisplaced fractures were often misclassified as no fracture, resulting in a low sensitivity of 51.2%. Mutasa et al.^15^ applied data augmentation techniques involving a generative adversarial network and a digitally reconstructed radiograph (DRR) to build an FNF classification model, which improved the AUC for fracture versus no fracture classification from 0.80 to 0.92. Similarly, femoral neck fractures were categorized as displaced or undisplaced, and the sensitivity of undisplaced fractures was 54% in the three-class classification including no fracture. Unlike our study, their ground truth labels for X-ray images were not established using 3D CT imaging, except for approximately 6% of cases used for DRR generation. Sato et al.^16^ developed a CAD system using a deep learning model for binary classification of hip fractures, achieving an accuracy of 96.1% and an AUC of 0.99. The CAD system helped mitigate discrepancies in diagnostic performance due to varying levels of clinical experience. Although Garden types in hip fractures were identified based on X-ray images, Garden type classification was not included in the model. Recently, González et al.^17^ developed segmentation and classification models utilizing YOLOv8 to analyze femoral fractures. The models categorized fractures into neck, pertrochanteric, and subtrochanteric regions, with FNF further divided into displaced and undisplaced types. They achieved a Dice similarity of 77% for segmentation and an accuracy of 86.2% for classification. Given the single-center design and the relatively small test set (105 subjects), the generalizability of the results may be limited.

Relatively lightweight models were employed in this study to ensure computational efficiency for timely inference and to reflect the practical constraints of clinical environments, where computing resources may be limited (Supplementary Table 5). Lightweight models minimize inference time, allowing rapid deployment even in emergency settings. Moreover, they facilitate integration with picture archiving and communication systems (PACS). In addition, we aimed to reduce the risk of overfitting, which commonly arises in binary classification tasks with limited dataset size. The availability of larger datasets would enable the deployment of large-scale architectures, such as Vision Transformers, which may achieve further performance improvements. Transfer learning has been widely validated as a more efficient strategy in most cases, even though training from scratch on large-scale datasets may achieve comparable performance. We also applied transfer learning using ImageNet and COCO pre-trained weights, considering both model performance and training efficiency. For data augmentation, we employed horizontal flipping only. In ablation testing, additional augmentations such as rotation, translation, and brightness adjustment did not produce a statistically significant improvement in performance. Nonetheless, incorporating more diverse augmentation strategies, such as elastic deformation technique or the use of synthetic data, may help enhance the generalizability of deep-learning models. These strategies may help address the limited availability of Garden type II fractures by effectively expanding the training data. Techniques such as cost-sensitive learning and oversampling such as SMOTE may further contribute to performance improvement in future work.

Recent studies have highlighted the concept of posterior tilt (posterior angulation) observed on lateral X-ray views. According to the study findings of Palm et al., a posterior tilt of ≥ 20° was the only predictor of the need for reoperation^18^. In other words, fractures that appeared undisplaced on AP X-ray views could ultimately be identified as actually being displaced. This explains why, in this study, the predictions using both AP and lateral views as input data were superior to those using only the AP view.

In South Korea, insurance reimbursement criteria established by the government are based on the Garden FNF type, leading to disputes, due to the low reliability of the X-ray-based Garden classification. With our algorithms, a classification accuracy similar to that of CT can be achieved by using only X-ray data, thereby reducing costs Furthermore, the proposed algorithm makes it possible to achieve effective treatment when using only X-ray images, even in nations where 3D-CT is not widely used for diagnosing FNFs, thereby significantly reducing social healthcare costs. This approach can also benefit patient health by reducing unnecessary radiation exposure from routine CT scans.

**Fig. 7:**
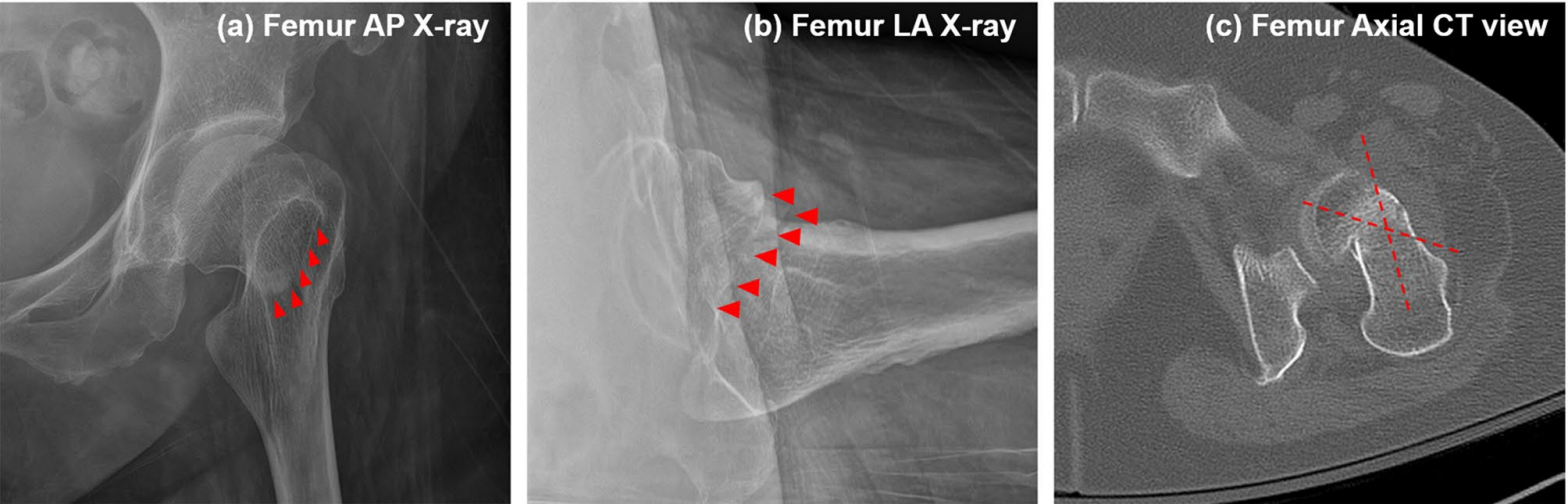
Example for misclassification of femoral neck fractures in X-ray images. Images of a 69-year-old female patient are presented: (a) X-ray anteroposterior (AP) view, (b) X-ray lateral (LA) view, and (c) axial computed tomography (CT) scan. The ensemble model classified the fracture as Garden type IV based on X-ray images of the AP and LA views. However, CT scan evaluation showed the fracture to be Garden type III due to posterior angulation.

The limitations of this study include the following. The ability of the deep-learning algorithm to distinguish between undisplaced fracture types (Garden types I and II) was not evaluated, mainly due to the limited number of Garden type II (*n* = 68). However, this is not clinically significant as the treatment for Garden types I and II fractures does not differ greatly. The accuracy in distinguishing between Garden types III and IV remains around 70%. While this accuracy level demonstrates value for screening purposes, the performance is insufficient to replace CT-based diagnosis. For example, as shown in Fig. 7, our ensemble model classified an FNF as Garden type IV based on X-ray views, whereas expert interpretation based on CT data identified it as Garden type III. This discrepancy highlights the model’s current limitations in terms of the ability to discern subtle posterior tilt or incomplete displacement when using only X-ray images. Notably, the Sankey diagram further emphasized that Garden type III fractures present significant classification challenges, warranting careful consideration of additional CT scans when such fractures are suspected. In clinical practice, the decision between osteosynthesis and arthroplasty in unstable fractures is primarily based on factors such as patient age, bone quality, and overall fracture stability, rather than the specific distinction between Garden type III and IV^19^. Furthermore, according to the NICE guidelines, Garden type I and II fractures, which account for approximately 5–15% of all femoral neck fractures, are recommended to be treated with osteosynthesis, while unstable fractures are generally managed with total hip replacement or hemiarthroplasty. Although Garden types III and IV are both classified as unstable fractures with only minimal differences in treatment strategies, improvements in classification accuracy are necessary to ensure sufficient reliability to replace CT-based diagnosis.

To enhance the generalizability and robustness of the proposed algorithm, further validation using multi-center datasets encompassing diverse patient populations, imaging equipment, and clinical practices is necessary. Moreover, prospective clinical studies are necessary to evaluate the prognostic value and real-world feasibility of the AI-based Garden type classification. Ultimately, cost-effectiveness analyses that take into account the clinical environments, healthcare infrastructure, and reimbursement systems of each country will be essential to assess the general applicability of our approach.

Despite these limitations in identifying each category of Garden classification, our deep-learning algorithm based on X-ray images demonstrated the potential to replace CT-based diagnosis. In addition, the confidence-based categorization in our approach may help identify patients who require CT-based diagnosis, allowing for more efficient allocation of imaging resources in clinical settings. For instance, CT evaluation may be recommended for patients with low confidence (< 95%) to assess fracture alignment, including posterior tilt (≥ 20°), which is a known high-risk predictor for re-operation. Furthermore, the proposed model could potentially be integrated with hospital PACS as a decision-support tool for emergency physicians, automatically flagging high-risk cases in real time. Further investigation across diverse patient populations, imaging equipment, and operators could expand the role of X-ray-based diagnosis for FNFs, offering a more cost-effective and convenient alternative for clinical settings.

## Supplementary Information

Below is the link to the electronic supplementary material.

Supplementary Material 1

## Data Availability

The data presented in this study are available on request from the corresponding author. The data are not publicly available due to conditions of the ethics committee of our university.
