# Supplementary material for: Garden classification of femoral neck fracture using deep-learning algorithm
Source: Sci Rep. 2025 Dec 16;15:43926. doi: 10.1038/s41598-025-27766-4 (PMC12708817; doi:10.1038/s41598-025-27766-4)

**Supplementary Table 1**. Training setup of deep learning models

| **Item** | **Detection model** | **Classification model** |
| --- | --- | --- |
| Input size | 800 × 800 | 256 × 256 |
| Pre-processing (normalization) | RescaleIntercept  WindowCenter  WindowWidth  Min/max nomalization  Zero-padding | RescaleIntercept  WindowCenter  WindowWidth  Min/max nomalization |
| Data augmentation | - | Horizontal flip |
| Batch size | 8 | 32 |
| Network | Faster R-CNN model with ResNet50-FPN backbone | EfficientNet (B0, B2, B4)  ResNet (18, 50, 101) ResNext50  RexNet (100, 130, 150)  DenseNet121  MobileNetV3 |
| Transfer learning | COCO | ImageNet |
| Optimizer | AdamW | AdamW |
| Number of epochs to train | 100 | 200 |
| Learning rate | 0.001 (fixed) | 0.001 (fixed) |
| Loss | RPN Loss (Binary Cross-Entropy Loss, Smooth L1 Loss) Fast RCNN Loss (Cross-Entropy Loss, Smooth L1 Loss) | Weighted Cross Entropy |

**Supplementary Table 2.** Comparison of model performance across five folds for the model using both anteroposterior (AP) and lateral (LA) views: undisplaced (Garden types I and II) versus displaced (Garden types III and IV) in internal dataset (N = 1,588). For individual networks, the best performance value for each metric is highlighted in bold. The ensemble results were obtained by combining the outputs of MobileNetV3, EfficientNetB4, and ResNet18, which were the top 3 models ranked by average DSC across five folds.

| Name | ACC (%) | | | | | AUC (%) | | | | | DSC (%) | | | | |
| --- | --- | --- | --- | --- | --- | --- | --- | --- | --- | --- | --- | --- | --- | --- | --- |
|  | Fold 1 | Fold 2 | Fold 3 | Fold 4 | Fold 5 | Fold 1 | Fold 2 | Fold 3 | Fold 4 | Fold 5 | Fold 1 | Fold 2 | Fold 3 | Fold 4 | Fold 5 |
| EfficientNetB0 | 88.4 | 90.6 | 88.7 | 84.9 | 89.5 | **94.4** | **96.1** | 93.6 | 90.2 | 94.4 | 86.0 | 88.6 | 86.6 | 81.0 | 87.2 |
| EfficientNetB2 | 89.7 | 88.1 | 90.3 | 87.1 | 86.9 | 93.5 | 92.7 | 94.6 | 91.6 | 93.5 | 87.4 | 85.4 | 88.1 | 84.1 | 83.8 |
| EfficientNetB4 | 88.7 | 89.7 | 90.6 | **88.3** | 88.5 | 94.3 | 93.6 | 95.5 | 91.0 | 93.9 | 86.3 | 87.3 | 89.1 | **85.4** | 85.8 |
| ResNet18 | 89.7 | 89.4 | 89.0 | 86.1 | 88.9 | 93.1 | 94.4 | 94.3 | 91.7 | 94.2 | **87.6** | 87.4 | 87.1 | 83.8 | 86.7 |
| ResNet50 | 89.0 | 89.7 | 90.6 | 85.8 | 87.3 | 93.1 | 95.3 | 95.7 | 92.0 | 95.0 | 87.1 | 87.6 | 88.5 | 82.2 | 84.4 |
| ResNet101 | 87.1 | 88.8 | 90.3 | 85.8 | **90.4** | 93.4 | 94.5 | 94.7 | 91.7 | 94.3 | 84.0 | 86.1 | 88.4 | 82.6 | 87.6 |
| ResNext50 | 89.0 | 90.0 | 88.1 | 87.4 | 88.5 | 92.6 | 95.2 | 94.9 | 92.3 | 94.0 | 86.6 | 87.9 | 85.2 | 85.1 | 86.2 |
| ReXNet100 | 89.0 | **92.2** | 85.5 | 86.4 | 89.8 | 93.3 | 95.8 | 92.1 | 92.2 | **95.1** | 86.7 | **90.7** | 82.8 | 84.3 | **87.9** |
| ReXNet130 | 87.5 | 90.0 | 90.3 | 86.4 | 87.6 | 92.8 | 95.8 | 94.7 | 91.2 | 93.0 | 84.7 | 87.3 | 88.3 | 84.2 | 84.2 |
| ReXNet150 | 88.4 | 87.8 | 89.6 | 86.8 | 89.2 | 93.9 | 95.5 | 95.2 | 92.4 | 94.3 | 85.6 | 84.4 | 88.1 | 83.8 | 86.4 |
| DenseNet121 | 86.8 | 84.7 | 89.6 | 87.4 | 87.6 | 94 | 92.7 | 95.2 | **92.7** | 94.2 | 82.6 | 80.1 | 87.3 | 84.6 | 85.2 |
| MobileNetV3 | **90.0** | 90.0 | **92.8** | 85.8 | 89.5 | 94.3 | 94.7 | **96.1** | 90.0 | 92.0 | **87.6** | 87.7 | **91.4** | 83.1 | 86.5 |
| Ensemble of top 3 | 90.6 | 91.2 | 91.8 | 89.0 | 90.1 | 94.8 | 95.5 | 96.3 | 92.0 | 96.0 | 88.6 | 89.4 | 90.4 | 86.6 | 87.9 |

ACC, accuracy; AUC, area under the curve; DSC, Dice similarity coefficient.

**Supplementary Table 3**. Classification performance of deep learning models for femoral neck fracture: Garden type III versus IV. These results represent the outcome of 5-fold cross-validation conducted exclusively on cases classified as Garden type III and IV fractures.

|  | **Overall** | | **Garden type lll** | | **Garden type lV** | |
| --- | --- | --- | --- | --- | --- | --- |
| **Name** | **ACC (%)** | **DSC (%)** | **Pr (%)** | **Re (%)** | **Pr (%)** | **Re (%)** |
| EfficientNetB0 | 67.3 | 65.6 | 62.8 | 53.5 | 69.8 | 77.3 |
| EfficientNetB2 | 68.3 | 66.8 | 63.7 | 56.0 | 71.0 | 77.1 |
| EfficientNetB4 | 68.9 | 67.2 | 65.2 | 54.9 | 70.9 | 78.9 |
| ResNet18 | 66.7 | 64.4 | 62.9 | 49.5 | 68.6 | 79.1 |
| ResNet50 | 68.7 | 67.6 | 63.1 | 60.2 | 72.3 | 74.7 |
| ResNet101 | 68.0 | 67.2 | 61.5 | 62.7 | 72.9 | 71.9 |
| ResNext50 | 68.9 | 67.7 | 63.8 | 59.1 | 72.1 | 75.9 |
| RexNet100 | 66.9 | 65.4 | 61.5 | 55.3 | 70.1 | 75.2 |
| RexNet130 | 67.7 | 66.3 | 62.4 | 57.0 | 71.0 | 75.3 |
| RexNet150 | 68.2 | 66.8 | 63.4 | 56.6 | 71.1 | 76.5 |
| DenseNet121 | 65.5 | 64.1 | 59.4 | 55.1 | 69.4 | 72.9 |
| MobileNetV3 | 67.1 | 65.8 | 61.5 | 56.8 | 70.6 | 74.4 |
| Ensemble 3 | 72.1 | 70.9 | 68.3 | 61.8 | 74.4 | 79.4 |

**Supplementary Table 4.** Comparison of model performance across five folds: undisplaced (Garden types I and II) versus displaced (Garden types III and IV) in external dataset (N = 100). For individual networks, the best performance value for each metric is highlighted in bold. The ensemble results were obtained by combining the outputs of MobileNetV3, EfficientNetB4, and ResNet18, which were the top 3 models ranked based on DSC in internal dataset.

| Name | ACC (%) | | | | | AUC (%) | | | | | DSC (%) | | | | |
| --- | --- | --- | --- | --- | --- | --- | --- | --- | --- | --- | --- | --- | --- | --- | --- |
|  | Fold 1 | Fold 2 | Fold 3 | Fold 4 | Fold 5 | Fold 1 | Fold 2 | Fold 3 | Fold 4 | Fold 5 | Fold 1 | Fold 2 | Fold 3 | Fold 4 | Fold 5 |
| EfficientNetB0 | 86 | 85 | 88 | 88 | 87 | 92.5 | 91.1 | 92.8 | 93.6 | 94 | 85.3 | 83.8 | 87.1 | 86.8 | 86.1 |
| EfficientNetB2 | **89** | 89 | 85 | 88 | 88 | 91.8 | 93.3 | 93 | 93.9 | 93.8 | 88.1 | 87.8 | 84.6 | 87.1 | 87.3 |
| EfficientNetB4 | **89** | 89 | 88 | 89 | 83 | 91.9 | 93.2 | **95.1** | 95.4 | 89.9 | 88.3 | 88.1 | 87.3 | 88.3 | 82.2 |
| ResNet18 | 85 | 87 | 86 | 85 | 84 | 89 | 91 | 92.6 | 92.1 | 91.6 | 84.3 | 85.8 | 85.3 | 84.2 | 83 |
| ResNet50 | 86 | **91** | **89** | 85 | 86 | 92.6 | **95** | 92 | 93 | 90.5 | 85.4 | **90.4** | 88.1 | 83.8 | 85.4 |
| ResNet101 | **89** | 82 | 88 | **91** | 84 | **93.6** | 88.6 | 92.6 | **96.8** | 93.2 | 88.3 | 80.7 | 87.1 | **90.3** | 82.6 |
| ResNext50 | 84 | 88 | 89 | 87 | 87 | 89.6 | 93.4 | 93.6 | 95.6 | **94.3** | 82.8 | 86.8 | **88.4** | 86 | 86.1 |
| ReXNet100 | **89** | 88 | 81 | 83 | 87 | 93.2 | 91.4 | 88.5 | 91.6 | 91.4 | **88.4** | 87.1 | 79.7 | 82.2 | 86.1 |
| ReXNet130 | 82 | 83 | **89** | 86 | 85 | 91.3 | 90.3 | 91 | 91 | 92.2 | 81.2 | 81.2 | 88.1 | 85.3 | 84 |
| ReXNet150 | 83 | 90 | 85 | 90 | **89** | 93.1 | 93.5 | 92.2 | 94.3 | 92.5 | 81.4 | 89.1 | 84.4 | 89 | **88.3** |
| DenseNet121 | 81 | 83 | 88 | 89 | 86 | 89.8 | 91 | 93.6 | 95.4 | 92.1 | 79.5 | 80.6 | 86.8 | 88.4 | 85.1 |
| MobileNetV3 | 86 | 86 | 87 | 88 | 85 | 93.6 | 92.9 | 93.4 | 91 | 89.8 | 84.4 | 85 | 86.4 | 87.4 | 83.8 |
| Ensemble of top 3 | 89 | 90 | 89 | 89 | 85 | 92.8 | 92.8 | 94.7 | 94.5 | 91.7 | 88.3 | 89.3 | 88.4 | 88.3 | 84.2 |

ACC, accuracy; AUC, area under the curve; DSC, Dice similarity coefficient.

**Supplementary Table 5.** Comparison of model complexity, computational cost, and performance across deep-learning architectures between CNN and visual transformer architectures.

| Models | Base model parameters (millions) | Model parameters (millions) | Vram (GB) | Training time (second/epoch) | Inference time (ms/image) | Validation accuracy | Test accuracy |
| --- | --- | --- | --- | --- | --- | --- | --- |
| mobilenetv3_large_100 | 5.48 | 12.61 | 3.58 | 8.89 | 3.22 | 92.19% | 88.75% |
| efficientnet_b0 | 5.29 | 12.03 | 6.13 | 12.41 | 5.20 | 91.88% | 88.44% |
| vit_medium_patch16_gap_256 | 38.86 | 115.05 | 8.31 | 24.97 | 8.37 | 73.75% | 68.75% |

**Supplementary Figure 1. Confusion matrix heatmaps of Garden type classification.**


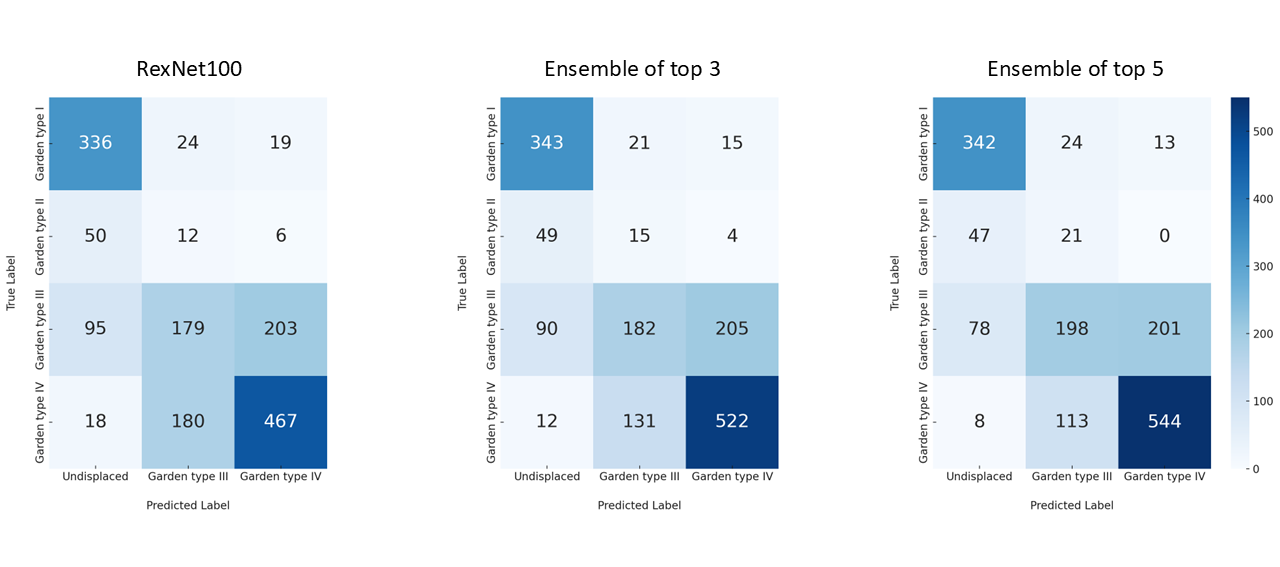

Supplement: Supplementary file 1 — Supplementary Material 1 [file 41598_2025_27766_MOESM1_ESM.docx]
